# Supplementary material for: Chemical Treatments on Invasive Bivalve, Corbicula fluminea
Source: Animals (Basel). 2024 Jun 14;14(12):1789. doi: 10.3390/ani14121789 (PMC11201199; doi:10.3390/ani14121789)
Supplement: Supplementary file 1 [file animals-14-01789-s001.zip › animals-3019866-supplementary.pdf]

Table S1. Geo-referenced records of *Corbicula fluminea* in Massachusetts, 1999-2023.

Site Number	First Year Reported	Water Body	Latitude	Longitude	Agency	Report Source
1	1999	Long Pond	-70.9494	41.8000	DCR	DCR Data from Brian Read 1999
2	2001	Charles River	-71.1816	42.3606	USGS	USGS 8/22/2016 http://nas.er.usgs.gov/default.aspx
3	2001	Tispaquin Pond	-70.8581	41.8636	DCR	DCR Data from J. Cordeiro 2001
4	2001	Charles River	-71.1355	42.3669	USGS	USGS 8/22/2016 http://nas.er.usgs.gov/default.aspx
5	2001	Charles River	-71.1555	42.3597	USGS	USGS 8/22/2016 http://nas.er.usgs.gov/default.aspx
6	2001	Charles River	-71.1324	42.3713	USGS	USGS 8/22/2016 http://nas.er.usgs.gov/default.aspx
7	2005	Congamond Lakes, Middle Basin	-72.7558	42.0243	USGS	USGS 8/22/2016 http://nas.er.usgs.gov/default.aspx
8	2005	Congamond Lakes, North Basin	-72.7575	42.0375	DCR	DCR Data from Lakes & Ponds 2005
9	2005	Congamond Lakes, South Basin	-72.7664	42.0221	DCR	DCR Data from Lakes & Ponds 2005
10	2005	Webster Lake	-71.8579	42.0584	USGS	USGS 8/22/2016 http://nas.er.usgs.gov/default.aspx
11	2005	Fort Meadow Reservoir	-71.5300	42.3750	USGS	USGS 8/22/2016 http://nas.er.usgs.gov/default.aspx
12	2006	East Brimfield Reservoir	-72.1327	42.1097	MassDCR	DCR: National Heritage 2006 DFW T. French
13	2007	Fivemile Pond	-72.5114	42.1421	MassDCR	DRC: Lakes &Ponds 2007
14	2007	Lake Sabbatia	-71.1093	41.9435	MassDCR	DCR: Lakes & Ponds 2007
15	2007	Winnecunnet Pond	-71.1313	41.9708	MassDCR	DCR: National Heritage 2007 E. Nedeau
16	2007	Oldham Pond	-70.8347	42.0667	MassDCR	DCR: J. Cordeiro 2007
17	2007	Sampson Pond	-70.7510	41.8501	MassDCR	DCR: Lakes & Ponds 2007
18	2007	Mashpee Pond	-70.4867	41.6611	MassDCR	DCR: National Heritage 2007 E. Nedeau
19	2007	Ashumet Pond	-70.5340	41.6335	DCR	DCR Data from Nat Heritage 2007-E. Nedeau
20	2008	Peters Pond	-70.4912	41.6889	DCR	DCR Data from Lakes and Ponds 2008
21	2010	Norton Reservoir	-71.1974	41.9837	USGS	USGS 8/22/2016 http://nas.er.usgs.gov/default.aspx
22	2010	Taunton River	-71.0751	41.9031	USGS	USGS 8/22/2016 http://nas.er.usgs.gov/default.aspx
23	2010	Taunton River	-71.0646	41.9034	USGS	USGS 8/22/2016 http://nas.er.usgs.gov/default.aspx
24	2010	Taunton River	-71.0298	41.8867	USGS	USGS 8/22/2016 http://nas.er.usgs.gov/default.aspx
25	2010	Taunton River	-70.9955	41.9034	USGS	USGS 8/22/2016 http://nas.er.usgs.gov/default.aspx
26	2010	Taunton River	-70.9428	41.9362	USGS	USGS 8/22/2016 http://nas.er.usgs.gov/default.aspx
27	2010	Taunton River	-71.0020	41.8939	USGS	USGS 8/22/2016 http://nas.er.usgs.gov/default.aspx
28	2010	Taunton River	-71.0474	41.9047	USGS	USGS 8/22/2016 http://nas.er.usgs.gov/default.aspx
29	2010	Middle Pond	-70.4156	41.6686	USGS	USGS 8/22/2016 http://nas.er.usgs.gov/default.aspx
30	2013	Bennett Pond	-72.4519	42.0914	Individual	Joy Trahan-Liptak
31	2013	Massapoag Lake	-71.1775	42.1035	Individual	Joy Traha, no agency
32	2013	Unnamed Pond South of Hodges Village Resevoir	-71.8778	42.1180	Individual	Joy Trahan-Liptak
33	2014	Unnamed Forest Park Tributary	-72.5656	42.0798	MassDEP	14-C001-05, 14-C008-05
34	2014	Nemasket River	-70.9424	41.9362	Individual	Joy Trahan-Liptak
35	2015	Blackstone River	-71.6171	42.0922	Individual	Joy Trahan-Liptak
36	2015	Spy Pond	-71.1528	42.4070	USGS	USGS 8/22/2016 http://nas.er.usgs.gov/default.aspx
37	2015	Hamblin Pond	-70.4105	41.6634	Individual	Personal communication 8/31/2016 Ken Wagner
38	2016	Concord River	-71.3360	42.4790	MassDEP	Personal communication 11/8/2016 Eileen McGourty, FWS
39	2016	Lake Cochituate: Small Middle Basin	-71.3704	42.3003	DCR	Personal communication, Tom Flannery 10/21/2015 DCR
40	2016	Lake Cochituate: Second Northern Basin	-71.3748	42.3103	DCR	Personal communication, Tom Flannery 10/21/2015 DCR
41	2016	Dudley Pond	-71.3733	42.3321	Wayland Surface Water Quality Committee	Personal communication, Mike Lowery, Wayland Surface Water Quality Committee, 10/24/2016
42	2016	Sudbury River	-71.3944	42.3353	FWS	Personal communication 9/6/2016 Eileen McGourty FWS
43	2017	Heart Pond	-71.3877	42.5657	MassDEP	Fish Servey May23th 2017
44	2018	Whitins Pond	-71.6905	42.1145	MassDEP	Therese Beaudoin
45	2018	Lake Quinsigamond	-71.7482	42.2568	MassDEP	Personal communication, 7/30/2018, Michael Brauer and David Wong
46	2020	Assawompset Pond	-70.9231	41.8371	DCR	Personal communication, 10/19/2020, Jim Straub and Karen Colby
47	2020	Field Pond	-71.1088	42.6082	DCR	Personal communication, 10/15/2020. Jim Straub and Ellie Jane
48	2021	Onota Lake	-73.2827	42.4700	DCR	Personal communication, 9/22/2021, Jim Straub, Jim McGrath
49	2022	Pontoosuc Lake	-73.2489	42.4955	MassDEP	Personal Communication, 12/8/2022, David Wong, Robert Van Der Kar
50	2023	Cheshire Resevoir	-73.1895	42.5347	MassDEP	Personal communication, 8/10/2023, Jim Straub, David Wong
51	2023	Pequot Pond	-72.6942	42.1860	DCR	Kara Sliwoski DCR
52	2023	Manhan River	-72.6543	42.2800	MassDEP	Steven Bittner DEP
53	2023	Lake Lorraine	-72.5136	42.1457	MassDEP	Steven Bittner DEP
54	2023	Neponset Reservoir	-71.2476	42.0857	MassDEP	Personal Communication, 3/22/2023, David Wong, Ken Wagner
